# Sensory Feedback in Interlimb Coordination: Contralateral Afferent Contribution to the Short-Latency Crossed Response during Human Walking

**DOI:** 10.1371/journal.pone.0168557

**Published:** 2017-01-06

**Authors:** Sabata Gervasio, Michael Voigt, Uwe G. Kersting, Dario Farina, Thomas Sinkjær, Natalie Mrachacz-Kersting

**Affiliations:** 1 Center for Sensory-Motor Interaction (SMI), Department of Health Science and Technology, Aalborg University, Aalborg, Denmark; 2 Department of Bioengineering, Imperial College London, London, UK; 3 Villum Fonden, Søborg, Denmark; Universite de Nantes, FRANCE

## Abstract

A constant coordination between the left and right leg is required to maintain stability during human locomotion, especially in a variable environment. The neural mechanisms underlying this interlimb coordination are not yet known. In animals, interneurons located within the spinal cord allow direct communication between the two sides without the need for the involvement of higher centers. These may also exist in humans since sensory feedback elicited by tibial nerve stimulation on one side (ipsilateral) can affect the muscles activation in the opposite side (contralateral), provoking short-latency crossed responses (SLCRs). The current study investigated whether contralateral afferent feedback contributes to the mechanism controlling the SLCR in human gastrocnemius muscle. Surface electromyogram, kinematic and kinetic data were recorded from subjects during normal walking and hybrid walking (with the legs moving in opposite directions). An inverse dynamics model was applied to estimate the gastrocnemius muscle proprioceptors’ firing rate. During normal walking, a significant correlation was observed between the magnitude of SLCRs and the estimated muscle spindle secondary afferent activity (P = 0.04). Moreover, estimated spindle secondary afferent and Golgi tendon organ activity were significantly different (P ≤ 0.01) when opposite responses have been observed, that is during normal (facilitation) and hybrid walking (inhibition) conditions. Contralateral sensory feedback, specifically spindle secondary afferents, likely plays a significant role in generating the SLCR. This observation has important implications for our understanding of what future research should be focusing on to optimize locomotor recovery in patient populations.

## Introduction

Following an unexpected perturbation to one limb (ipsilateral) a reflex response is often observed in the opposite limb (contralateral); this response, referred to as a crossed reflex, is likely mediated by commissural interneurons that directly connect muscles of opposing limbs within the animal spinal cord [1–3]. Animal research on crossed spinal reflexes documented the occurrence of a reversal from crossed extensor to crossed flexor response and vice versa [4–7]. The evidence provided by these studies suggests that the factor determining whether a crossed extensor or flexor response is elicited, is the position of the contralateral limb [4–7], such that the responses are directed toward the muscle that is more stretched [4,5,8]. For instance, spinal cats treated with clonidine, a noradrenergic receptor stimulant that reverses spinal cord injury paralysis in the cat [9], showed a reversal from crossed flexion to extension when the position of the contralateral hind limb was changed from being extended to being flexed [6,7]. A reversal from extensor to flexor responses has also been observed in intact walking cats when stimuli were delivered at the end of the contralateral stance phase, when the limb is extended [10]. After tenotomy of both antagonist muscles, the responses were still present, which suggests that the other intact pairs of muscles acting on the same joint also contribute to the generation of the response [6,7]. The most commonly evoked response seems to be crossed extension, unless flexor muscles are stretched; in the latter case a crossed flexion is evoked. Complete contralateral deafferentation (following dorsal rhizotomy), removed crossed flexor but not crossed extensor responses [7]. Contralateral muscle receptors have been suggested to have a predominant role of in the control of this crossed reflex reversal in animals [11].

Recent studies [12–15] suggest that commissural interneurons might also exist in the human spinal cord. Crossed inhibitory responses have been elicited in the soleus (SOL) muscles with a latency (40 ms after ipsilateral tibial nerve (iTN) stimulation) suggesting that these responses are spinally mediated [14]. Crossed excitatory responses in the gastrocnemius muscles show a longer latency (69 ms after iTN stimulation) and are reverted to an inhibition according to the task demands [15]. The magnitude of the SLCR has been previously compared between the end of the ipsilateral swing (80% of the ipsilateral gait cycle) during normal walking and the end of the ipsilateral stance phase (50% of the ipsilateral gait cycle) during hybrid walking (with the ipsilateral leg coordinated as moving forward, stepping on a belt of treadmill moving backward, and with the contralateral leg coordinated as moving backward, stepping on a belt of the treadmill moving forward) [15]. These two timings were selected since they show comparable ankle joint angles and comparable activation levels in the triceps surae. Moreover, both timings correspond to a transition phase, from ipsilateral swing to stance in normal walking and from stance to swing during hybrid walking. The instability of the phase would require appropriate interlimb coordination in order to react rapidly to a disturbance of balance [15], although the tasks have different functional demands. During normal walking, when the iTN stimulation is delivered at 80% of the ipsilateral gait cycle, the inhibition of the contralateral SOL (cSOL) and the facilitation of the contralateral gastrocnemius might accelerate the leg into initiating swing of the contralateral leg, in the event that the ipsilateral leg is not able to support body weight [16]. A deviation of the center of pressure trajectory toward the medial lateral direction under the contralateral foot was indeed observed following the response and this was accompanied by reduction of stance phase duration for the ispilateral foot immediately after the stimulation [16]. During hybrid walking, when the iTN is delivered at 50% of the ipsilateral gait cycle, a crossed inhibition is observed in the gastrocnemius. At this timing, the ipsilateral leg is preparing to push off while the contralateral leg is touching down. In this situation, the inhibition of the gastrocnemius assisting the extension of the knee and forcing the heel to the ground, would be the most appropriate reaction [15].

**Fig 1:**
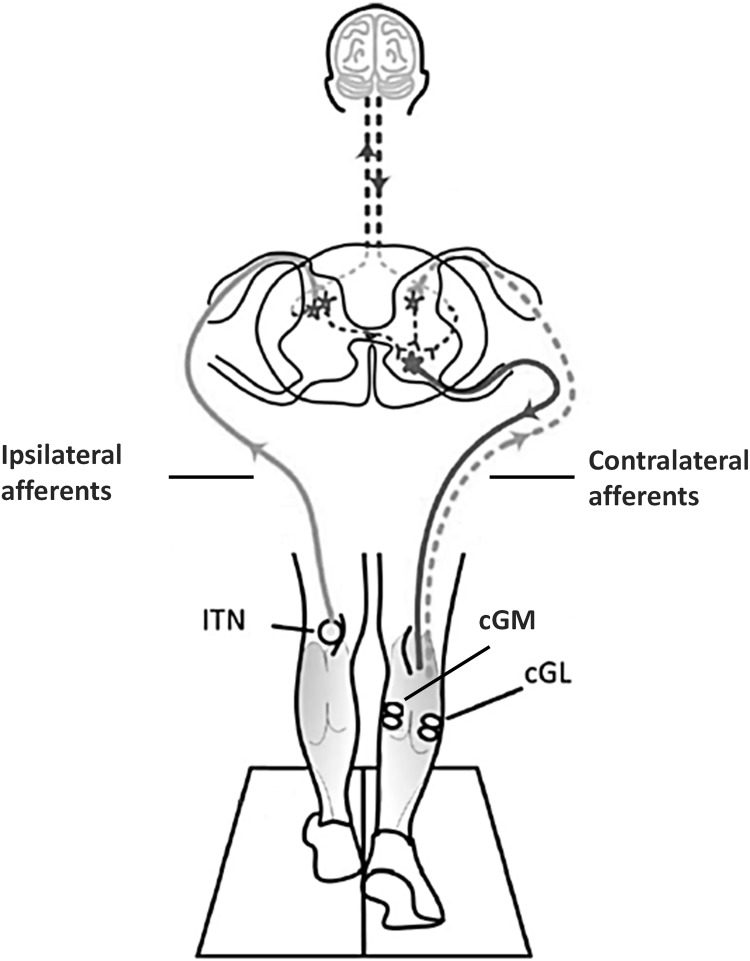
Neural pathways likely involved in the generation of the SLCR. SLCRs are elicited in the contralateral gastrocnemius medialis (cGM) and lateralis (cGL) following stimulation of the tibial nerve (iTN) in the ipsilateral leg during human walking. The current study was designed to investigate whether the activity of contralateral afferents (dashed gray lines) contributes to the SLCR. The gray and black full lines represent the ipsilateral afferent pathways and contralateral efferent pathways involved in the generation of the SLCR, respectively. Supraspinal contribution to the SLCR is not excluded (dashed black line). All other dashed lines in the spinal cord represent unknown pathways.

The aim of the current study was to elucidate the mechanism behind the generation of the SLCR investigating whether the activity of contralateral afferents contributes to the SLCRs (Fig 1). Gastrocnemius muscle afferent activity (group Ia, Ib, and II afferent activity) was estimated using a combination of three models described in Voigt et al. [17] and Prochazka and Gorazini [18] and Mileusnic and Loeb [19], respectively, parametrizised with human in vitro and in vivo anatomical and mechanical data and chronic recordings of afferent activity in cats. A mathematical model has been preferred over the use of ultrasound scanning techniques; since fascicle length changes obtained with ultrasonography do not provide a ‘golden standard’ [20], we assume that using the tendon model [17] to obtain muscle fascicle length changes gives an equally good and qualified estimate. Since animal literature suggests that contralateral receptors from muscles acting on the same joint [6,7] have a predominant role in the control of crossed reflex reversal in animals [11], with the responses directed toward the muscle that is more stretched [4,5,8], we hypothesize that the modulation of the SLCR is correlated with the estimated group II afferent activity of both the contralateral gastrocnemius medialis (cGM) and lateralis (cGL). Accordingly, we also hypothesize that the reversal in the response observed between normal walking at 80% of the gait cycle and hybrid walking at 50% of the gait cycle [15], is related to differences in the estimated contralateral afferent activity between these two conditions.

## Materials and Methods

**Fig 2:**
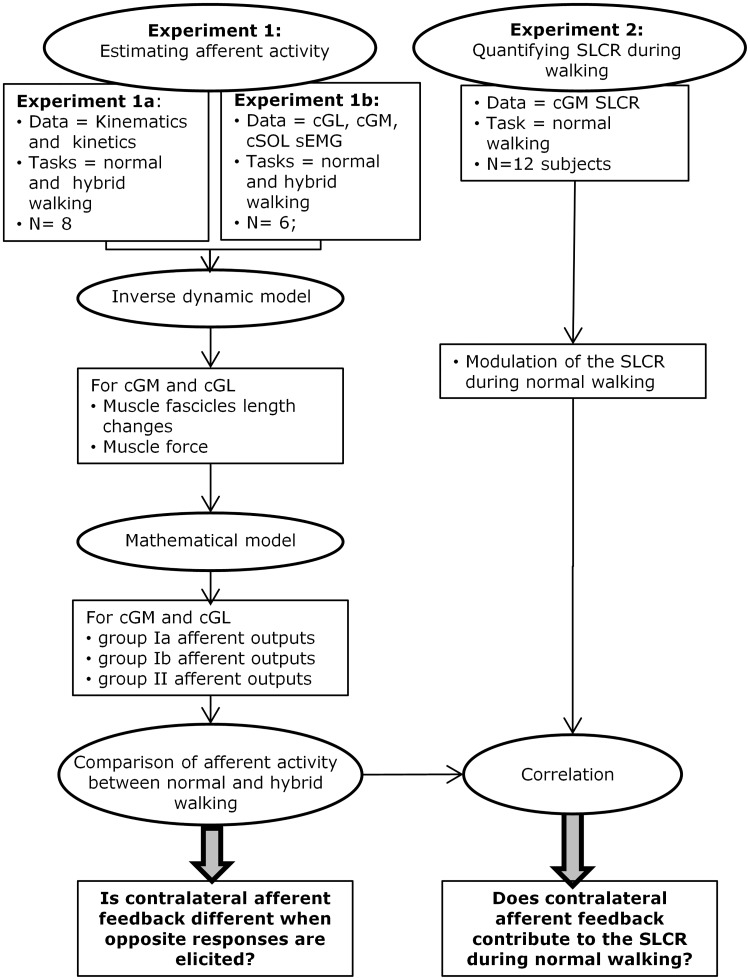
Overview of the experiment and the analysis performed. The aim of experiment 1 was to estimate muscle afferent output for the two heads of the contralateral gastrocnemius. In experiment 1a, kinematic and kinetic data was collected from 8 subjects during normal and hybrid walking. In experiment 1b, muscle activation during normal and hybrid walking was recorded from the triceps surae of 6 subjects to evaluate muscle relative activation. Data from Experiments 1a and 1b was combined to estimate, using an inverse dynamics model modified from [17], muscle fascicles length changes and muscle force for the two heads of the gastrocnemius. A mathematical model modified from [18,19] was then used to estimate group Ia, Ib and II afferent outputs. The estimated contralateral afferent activity was compared between these normal (80% gait cycle) and hybrid walking (50% gait cycle) conditions to assess whether a different afferent activity is accountable for the reversal in the response. In experiment 2, the modulation of the SLCR was quantified in 12 subjects during normal walking. The modulation of the response was then compared with the estimated activity of the different afferents to evaluate whether contralateral afferent activity contributes to the generation of the SLCR.

Twenty-six subjects took part to the experiments. None of the participants was affected by any central or peripheral movement disorders. The participants provided their written informed consent to the protocol approved by the Scientific Ethics Committee of Nord-Jutland (approval number: N-20090037, N-20110040) as required by the standards of the Declaration of Helsinki. The study comprised two experiments (Fig 2). Experiment 1 was designed to estimate muscle Golgi tendon organ (GTO) activity (group Ib afferent output) and spindle activity (group Ia and II afferent outputs) for the two heads of the contralateral gastrocnemius during normal and hybrid walking. In the first part of the experiment, experiment 1a, kinematic and kinetic data was collected from 8 subjects during normal and hybrid walking. During the second part of the experiment, experiment 1b, muscle activation during normal and hybrid walking was recorded from the triceps surae of 6 subjects to evaluate muscle relative activation. Data from Experiments 1a and 1b was used to estimate muscle fascicles length changes and muscle force for the two heads of the gastrocnemius through an inverse dynamics model modified from [17]. A mathematical model modified from [18,19] was then used to estimate group Ia, Ib and II afferent outputs. This method was used as current techniques do not allow to directly measure ensemble afferent activity in humans in vivo. Since the SLCR has been shown to be facilitatory during normal walking (at 80% of the ipsilateral gait cycle) and inhibitory during hybrid walking (at 50% of the ipsilateral gait cycle) [15] the estimated contralateral afferent activity was compared between these two conditions to assess whether a different afferent activity is accountable for the reversal in the response. The aim of experiment 2, to which 12 subjects participated, was to describe the existence of a SLCR in the cGM and quantify the modulation of such a response during normal walking in order to compare it with the estimated activity of the different afferents.

### General experimental protocol

**Fig 3:**
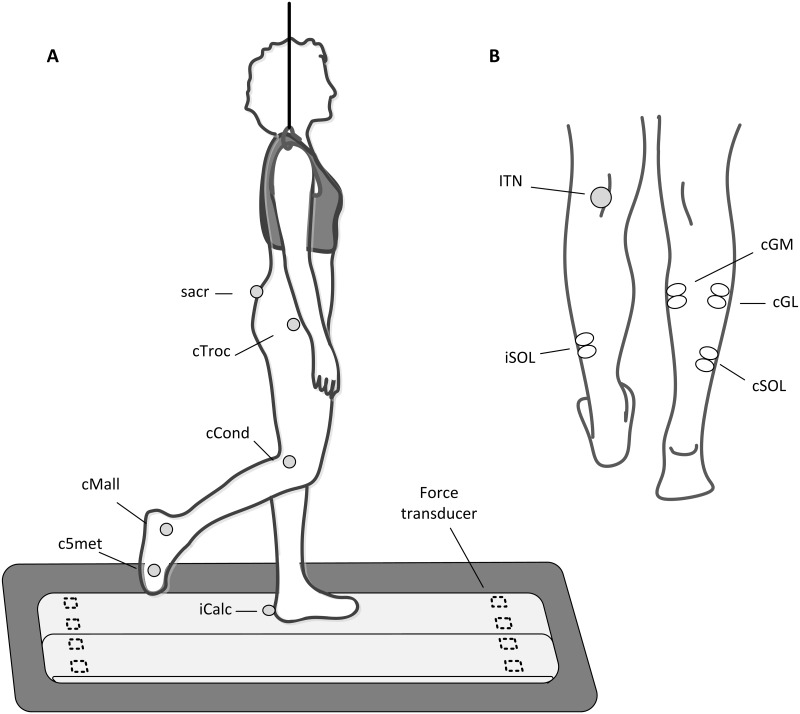
Experimental set up. A: The subjects walked on a split belt treadmill. The vertical component of the GRF was recorded through 8 force transducers placed at the corners under each belt. Subjects wore a safety harness that did not alter the subjects’ body weight support. Retroreflective ball-shaped markers were placed on the skin of the subject’s over the ipsilateral calcaneus (iCalc) and on contralateral leg over the following anatomical landmarks: fifth metatarsal joint (c5met), on the lateral malleolus (cMall), on the lateral epicondyle of the femur (cCond), on the great trochanter (cTroc) and on the sacral bone (sacr). B: Crossed responses in cGM were investigated after iTN stimulation. Bilateral SOL and cGL sEMG signal were also collected in order to evaluate the correct stimulation location and intensity (iSOL) and the relative activation of triceps surae muscles for the estimation of the force produced by the individual muscles (cSOL, cGM and cGL).

The subjects were asked to walk on an instrumented split belt treadmill (Woodway GmbH, Weil am Rhein, Germany) that allowed the two belts to be controlled independently. Subjects wore a harness for safety purposes that did not alter the subjects’ body weight support (Fig 3A). Stride time was defined as the time between two consecutive touch downs of the ipsilateral heel and the gait cycle percentage was defined so that 0% corresponded to the ipsilateral touch down and 100% corresponded to the next ipsilateral touch down. To limit the inter-step variability, the subjects were asked to maintain the same cadence throughout the testing period and were provided with verbal feedback whenever their stride times deviated. A self-selected speed was adopted since previous research documented an enhanced stability and adaptability of the gait cycle at the preferred speed [21]. Subjects selected their natural walking speed during 2–3 minutes of familiarization while walking on the treadmill, before the data collection commenced. Stride times were successively analyzed off-line and strides differing from the mean gait cycle duration by more than 10% were excluded from the analysis. This was done to make sure that the responses were evoked in the desired timings as SLCR were elicited by electrical stimulation delivered at specific percentage of the gait cycle duration. The subjects were allowed to take a break or stop at any time.

#### Electrical stimulation and sEMG

Surface electromyography (sEMG) and electrical stimulation were used in experiment 1b, for quantifying the relative activation of the cSOL, cGL and cGM (see ***Experiment 1*. *Estimation of afferent activity***, *Estimation of muscle spindle and GTO activity*), and in experiment 2 for quantifying the crossed response in cGM. An isolated stimulator (Noxitest IES 230, Aalborg, Denmark) was used for iTN stimulation with single monopolar stimuli of 1 ms duration. The stimulating electrodes were placed while standing; the cathode (PALs Platinum round electrode, Model No. 879100, Axelgaard Man, Lystrup, Denmark) was placed over the popliteal fossa (Fig 3B) and the anode (PALs Platinum rectangular electrode, Model No. 895240, Axelgaard Man, Lystrup, Denmark) at the anterior aspect of the knee joint just above the patella. In order to find the optimal location for the electrode placement, stimuli were delivered every 4 to 7 s and the cathode’s position was adjusted until a stable M-wave was observed, with minimal contamination from the stimulation artifact, in the ipsilateral SOL muscle (iSOL) muscle. sEMG was recorded with a single differential configuration following appropriate skin preparation and using single use surface electrodes (Neuroline 720 silver/silver-chloride, AMBU A/S, Denmark). Electrodes were placed on the SOL muscle of both legs and on cGL and cGM (Fig 3B), in accordance with the recommendations of [22] for SOL and with the SENIAM recommendations for GL and GM [23]. A reference electrode was placed over the tibial bone. Data were acquired using the Labview based acquisition tool Mr. Kick II 2.3 (Knud Larsen, Center for Sensory-Motor Interaction, Aalborg University, Denmark). Surface EMGs were pre-amplified (x100) and sampled at 2 kHz. All data were analyzed off-line using the computing environment MATLAB R2010b (MathWorks). Signals were band-pass filtered between 25 and 400 Hz, rectified, low pass filtered with a cut-off frequency of 40Hz, if not stated otherwise, and averaged (around 30 envelopes) to obtain an average gait cycle for each subject.

### Experiment 1. Estimation of afferent activity

#### Data acquisition

Eight age and height matched female subjects participated in the experiment 1a: age 24 ± 1 years, height 1.68 ± 0.13 m, body weight 62.4 ± 9.2 kg, shank length 0.41 ± 0.02 m (mean ± SD). Kinematic and kinetic data were acquired during one minute of normal and hybrid walking. For the hybrid walking condition, the direction of the belt under the contralateral leg was inverted, causing the subject to walk forward with the ipsilateral leg and backward with the contralateral leg, yet maintaining the same belt speed as for normal walking. The subjects were given 15 minutes to familiarize themselves with the hybrid walking task before the data collection started. This familiarization period allowed the subjects to reduce stride time variability due to the adaptation process [15]. Retroreflective ball-shaped markers were placed on the skin of the subject, over the ipsilateral calcaneus and on the contralateral leg over the following anatomical landmarks: fifth metatarsal joint, the tip of the lateral malleolus, the lateral epicondyle of the femur, the great trochanter and the sacral bone (Fig 2A) [17]. All participants walked barefoot to allow a better tracking of anatomical landmarks. The markers’ position was tracked with a motion analysis system with eight infrared digital video cameras (ProReflex MCU 240, Qualisys, Gothenburgh, Sweden) with a sampling frequency of 240 Hz. QTM Qualisys Track Manager version 2.5 software was used for data acquisition. The vertical component of the ground reaction force (GRF) on the belts was measured by eight built in force transducers (quartz force-sensing elements, type 9321B, Kistler Instrument Corp., USA). The placement of the force transducers, at the corners under each belt, allows GRF for each leg to be recorded separately. Kinetic data were acquired at 1200 Hz together with the kinematic data. Subjects walked placing each foot on the respective belt in order to collect the GRF produced by each foot separately. This condition slightly deviated from the natural gait pattern as under normal walking conditions foot placement occurs at midline. However, after a short adaptation period subjects reported feeling comfortable with the new walking pattern.

#### Data processing

**Fig 4:**
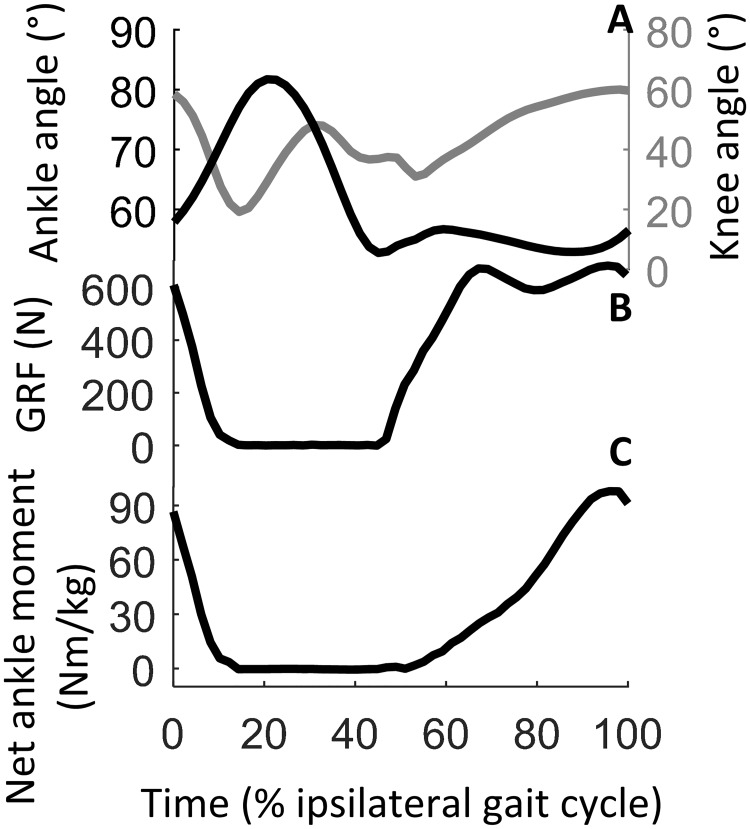
Kinetic and kinematic data used to calculate the net ankle moment during normal walking. All data represent an average gait cycle for one subject. A. Ankle joint (black) and knee joint (gray) kinematics. B. Vertical component of the GRF for the contralateral foot. C. Estimated net ankle moment for the contralateral leg.

Kinematic data were low-pass filtered with a 6 Hz cut-off frequency. Kinematic and kinetic data were segmented for each stride, interpolated at 50 samples in order to obtain the same number of samples for all gait cycle, and averaged in order to obtain an average gait cycle time for each subject (Fig 4A and 4B, S1 file). Heel strike was identified as the time in which the vertical position of the heel marker reached its lowest spot for each gait cycle. Inverse dynamic analysis was applied on a four link segment two dimensional model of the leg [17,24]. Ankle and knee joint angles and joint angular velocities were calculated from averaged gait cycle coordinates for each subject. Since the treadmill only allowed acquisition of the vertical component of the GRF (Fig 4B), the horizontal component was estimated from the subject’s body weight and the acceleration of the center of body mass; the latter was obtained from the kinematic data [25] of the sacral bone marker [26] as during walking the center of gravity is located in the region just anterior to the top of the second sacral segment [27]. Center of Pressure (CoP) coordinates were derived from the force measures, knowing the position of force transducers under the treadmills belts, and used to compute the net ankle moment (Fig 4C).

#### Estimation of muscle spindle and GTO activity

The estimation of the afferent output from the muscle spindles was based on the assumption that the muscle spindles are located in parallel with the muscle fibers. Muscle fascicle length changes were computed as the difference between the origin to insertion length change and the tendon length change [17]. To take into account the pennation of the muscle fibers, the muscle fascicle length changes were estimated by dividing the length obtained considering the fascicles parallel to the tendon, with the cosine of the pennation angle for the cGM. The latter parameter was extracted from the data of [28]. Since the current literature does not provide any data about the pennation angle for cGL during walking, the ratio between the pennation angle of cGM and cGL was derived from data reported during active and passive knee and ankle angle displacements [29] (S2 file). A 2-way Anova was used to evaluate differences in cGM/cGL pennation ratio at different knee and ankle positions, while a 1-way Anova was used to evaluate differences in cGM/cGL pennation ratio between active and passive conditions. No significant differences were found between cGM/cGL pennation ratio at different knee positions (0° and 45°) and ankle position (-15°, 0°, 15°, 30°) (F_(7,8)_ = 1.10, P = 0.45, 2-way Anova) and between passive and active conditions (F_(1,14)_ = 0.40, P = 0.54, 1-way Anova). Therefore, the pennation angle for cGL was derived from the pennation of cGM taking into account that the mean value for the cGM/cGL pennation ratio is 1.97. This value is also in accord with measures on cadavers [30–33]. During the hybrid walking task, since at 50% of the gait cycle angles of 71.5 ± 9.8° and 45.8 ± 7.2° (mean ± SD) were measured for the ankle and knee joints respectively, a pennation angle of 13° was adopted for the cGL since this pennation has previously been reported for the aforementioned joint angles [29].

Origin to insertion length changes were obtained through the following transfer functions from the joint angle [34] (Eqs 1 and 2).

$$\Delta Lgas_{i}=-22.18468+0.30141\theta_{i}-0.00061{\left(\theta_{i}\right)}^{2}+6.46251-0.07987\varphi_{i}+0.00011{\left(\varphi_{i}\right)}^{2}$$ (1)

$$\Delta Lsol_{i}=6.46251-0.07987\varphi_{i}+0.00011{\left(\varphi_{i}\right)}^{2}$$ (2)

*ΔLgas*_*i*_ is the length change of gastrocnemius associated with the knee angle (*θ*_*i*_) and ankle angle (*φ*_*i*_) while *ΔLsol*_*i*_ is the length change of the soleus associate with the ankle angle (*φ*_*i*_). The tendon length changes were attained from a non-linear tendon model [17] (Eqs 3 and 4) based on the calculation of the tendon force and including the following parameters: Young’s modulus (Y) for the tendinous tissue, the cross sectional area of the tendon (A_T_), the maximum tolerable strain for the tendon tissue before permanent damage (ε_max_), the length of the non-linear part of the stress-strain function (ε_T_), and the tendon resting length (l_0_) [17].

$$\Delta L_{T}=\sqrt{\frac{F_{T}}{k}}$$ (3)

where

$$k=\frac{YA_{T}(\varepsilon_{max}-\varepsilon_{T})10^{2}}{{\left(\varepsilon_{max}l_{0}\right)}^{2}}$$ (4)

The Achilles tendon force was obtained by dividing the net ankle moment by the moment arm. This moment arm was calculated by differentiating the origin to insertion length changes of SOL with respect to the ankle joint angle. The force produced by the individual muscles of the triceps surae, (SOL, GL and GM) depends on their physiological cross-sectional areas (PCSAs) and their relative activation levels. In experiment 1b, Average rectified sEMG amplitudes for cGL, cGM and cSOL were recoded from six subjects (4 females, age 29 ± 3 years) during normal and hybrid walking (see *Electrical stimulation and sEMG* for information regarding sEMG signal acquisition). The activation level for cSOL was recorded as this muscle also inserts into the Achilles tendon. After the initial band pass filtering, the EMG signals were rectified and low pass filtered with a 10 Hz cut-off frequency to obtain a linear envelope EMG and subsequently normalized to the respective maximum peak to peak direct motor response (M-max) [35] identified during standing, for each muscle (see *Electrical stimulation and sEMG* for description of electrical stimulation procedure). Activation levels of the three muscles were compared at three different times during the stance phase, namely at 70, 80, 90% of the gait cycle during normal walking and at 40, 50, 60% of the gait cycle during hybrid walking using a 2-way Anova. As no significant interaction was found between muscles and the timings (F_(4,45)_ = 0.42, P = 0.79, 2-way Anova) a 1-way Anova was performed on activation levels of cSOL, cGM and cGM for all sets of data. No difference was found (F_(2,51)_ = 1.40, P = 0.26); Achilles tendon force was therefore distributed in relation to the PCSAs of the muscles, namely 26% and 12% of the total cross-sectional area of triceps surae for cGM and cGL respectively [36]. Y and A_T_ were assigned the values of 1.7 GPA [37] and 46 mm^2^ respectively, as these values have been reported for a similar subject population as took part in the current study. The remaining model parameters were assigned the same values as in [17] that is 6% for the maximal tolerable strain for the tendon tissue before permanent damage ε_max_, 2% for the length of the non-linear part of the stress-strain function ε_T_ and 0.264m and 0.411m for the resting length l_0_ of tendinous structures for soleus and gastrocnemius, respectively.

In order to estimate the ensemble activity for muscle spindles primary and secondary afferent fibers, mathematical models previously used to predict spindles firing of mammalian triceps surae were adopted [18]. For spindle primary afferents activity (Ia), the following model was used:

$$Ia=4.3v^{0.6}+2l+b+f(EMG)$$ (5)

where *l* is the muscle fascicles length changes, *v* is the velocity of length changes, b is an offset value and f(EMG) is a signal representing the EMG-linked fusimotor action, obtained with the following function

f=EMG120(s+1)/(s+20 (6)

where EMG is the rectified, averaged and normalized sEMG signal for the investigated muscle and s is the Laplace operator.

To estimate spindles secondary afferents activity (II), the following model was used:

II=13.5l+b+20EMG (7)

where the last components is a signal representing the EMG-linked fusimotor action, obtained by scaling up the rectified, averaged and normalized sEMG of the investigated muscle by a factor of 20. These mathematical models were selected as they have been shown to provide the best fit to experimental data. The offset parameters *b* were assigned the value of 285 for muscle spindles primary afferents and 190 for muscle spindles secondary afferents in order to take into account that spindles afferents are never completely silent during a gait cycle [18]. However, the choice of different values for *b* would not have affected the statistical results of the current paper. In order to estimate the ensemble afferent activity from single fibers firing, the number of muscle spindles in human gastrocnemius muscle, that is 156 muscle spindles [38], was taken into account.

GTO activity was evaluated by deriving the estimated ensemble GTO activity (Ib) from the muscle force (F), using the following relationship obtained for mammalian gastrocnemius [19]:

$$Ib=10^{0.4939log_{10}\left(F\right)+3.2154}.$$ (8)

The average estimated afferent activity across all subjects was computed and expressed in pulses per second (pps).

### Experiment 2. Quantification of the modulation of SLCR

#### Data acquisition

Twelve subjects (6 females, age 24 ± 3 years) participated in experiment 2, where the modulation of the amplitude of cGM crossed responses during the gait cycle was quantified at 10 different times. sEMG was recorded from the iSOL and from the cGM. Foot switch sensors were placed under the ipsilateral heel, in order to determine the stride times. All signals were sampled at 2 kHz and stored for off-line analysis.

Once the subject started walking on the treadmill at their self-selected pace, the average gait cycle duration was measured as the average stride time of 30 steps. Stimulation was then delivered at the selected times (0, 10, 20, 30, 40, 50, 60, 70, 80, 90% gait cycle) at an intensity of 85% of the of the iSOL respective M-max [13,15] (for stimulation procedure see *Electrical stimulation and sEMG*). Data for a gait cycle were recorded every 2–4 steps and, in order to create a control case, gait cycles in which a stimulation was delivered were alternated randomly with gait cycles with no stimulation. Data from 30 gait cycles were recorded for the stimulation condition at each of the stimulatation times (300 gait cycles) and for the control condition of “no stimulation”.

#### Data analysis

Following a preliminary inspection with the acquisition software Mr. Kick II 2.3 (Center for Sensory-Motor Interaction, Aalborg University, Denmark), data were exported and processed with MATLAB R2010b. To evaluate the magnitude of the response, the root mean square value (RMS) from the stimulated gait cycle was computed in a selected time window and expressed as a percentage of the RMS of the control gait cycle in the same window. Since the cGL crossed responses had a latency of 69.6 (mean) ± 9.3 (SD) ms and a peak at 81.1 (mean) ± 6.6 (SD) ms after the stimulation [15], a time window from 60 to 90 ms after the stimulation was selected for the quantification of the response in cGM. However, to assess whether the crossed response in this muscle has a different latency, the onset, peak and duration of a possible crossed response were investigated at the time, during the gait cycle, at which iTN stimulation has been shown to elicit the most prominent facilitation in the cGL, that is 80% of the gait cycle [15]. The onset was evaluated as the time in which the averaged sEMG associated to the stimulated gait cycle exceeded the value of the averaged control gait cycle for an amount of two times the standard deviation of the averaged control gait cycle computed in a time window of 20 to 100 ms after the stimulation. The duration of the response was evaluated as the time at which the averaged stimulation gait cycle remained above this threshold. The response was discarded if its duration was shorter than 10 ms.

### Statistics

To evaluate whether significant SLCRs were elicited the cGM, the magnitude of the response was analyzed for each timing during the gait cycle using a one-sample t-test. The modulation of the magnitude of response during the gait cycle was evaluated using a one-way repeated- measures ANOVA with as factor the timing of the gait cycle. To establish the location of the difference, a post hoc pairwise comparison with Bonferroni correction was used.

Differences in group Ia, Ib and II afferent output between normal walking at 80% of the gait cycle and hybrid walking at 50% of the gait cycle were analyzed using a paired samples *t*-test. Spearman correlation coefficients were computed to assess the relation between the amplitude modulation of the cGM responses and the group Ia, Ib and II afferent output of cGM and cGL. Spearman correlation was used as the nature of the relation is unknown and therefore it cannot be assumed that the two quantities have linear relation. Data from the population averages have been used for the correlations. Since animal studies suggested that crossed flexion is evoked when the flexor muscles are stretched, otherwise a crossed extension would be evoked [7], only afferent activity associated with a state of stretch of the muscle was compared to the magnitude of the response elicited in cGM. The muscle was considered in a state of stretch during its lengthening, thus from a plantarflexion to dorsiflexion. This occurred from 20 to 40% and from 60 to 90% of the ipsilateral gait cycle. P values were computed using Student's t distribution for a transformation of the correlation. Results were considered significant when P was less than 0.05. If not stated otherwise, all data are given as mean ± SD.

## Results

The results obtained with the described model are consistent with those reported by recent studies for human locomotion: the modulation of the estimated muscle fascicle length changes is in accord with those displayed by [28,39] while muscle force amplitude is consistent with that reported by [40], with a maximum force exerted during walking by the gastrocnemius lateralis being just above 200 N.

### Estimated muscle spindle and GTO activity during normal and hybrid walking

**Fig 5:**
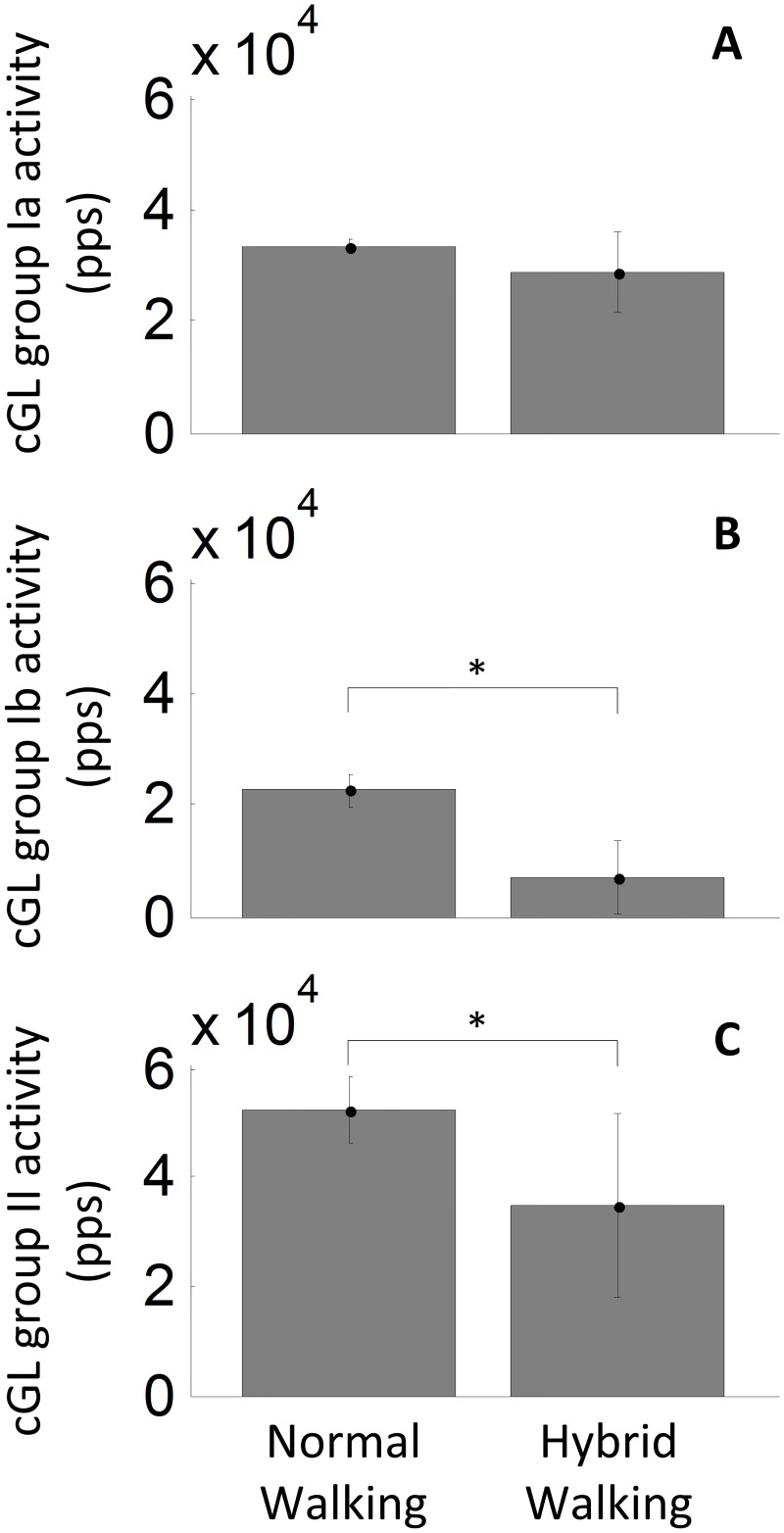
Differences in estimated afferent activity between normal and hybrid walking. Estimated ensemble activity for group Ia (A), Ib (B) and II (C) cGL’s afferent fibers at 80% of the normal walking ipsilateral gait cycle (left) and at 50% of the hybrid walking ipsilateral gait cycle (right). The data, collected from 8 subjects, are displayed as mean ± SD. * indicates significant differences.

Fig 5 displays the group Ia (A), group Ib (B) and group II (C) estimated activity for the cGL at 80% of the gait cycle during normal walking (on the left) and at 50% of the gait cycle during hybrid walking (on the right) (S3 file). The model estimates that in the normal walking condition, muscle fascicles are 9 mm longer and are stretching 1.8 mm/s slower than during the hybrid walking condition at the investigated timings. In addition, the force produced by the cGL was 225 N stronger at 80% of the gait cycle of normal walking compared to the 50% gait cycle of hybrid walking. These conditions yield to a similar estimated ensemble activity of muscle spindles primary afferents between the two tasks (*t*_(7)_ = 1.66, P = 0.14) with an estimated afferent activity of 33.3·10^3^ ± 1.1·10^3^ pps during normal walking and 28.8·10^3^ ± 7.3·10^3^ pps during hybrid walking (Fig 3A). Contrarily, GTO estimated activity was significantly different between the two tasks (*t*_(7)_ = 7.97, P < 0.001) at the time in which the responses were quantified [15], showing an ensemble estimated afferent activity of 22.6·10^3^ ± 3.1·10^3^ pps during normal walking and of 6.8·10^3^ ± 6.7·10^3^ pps during hybrid walking (Fig 3B). The estimated ensemble activity for muscle spindles secondary afferents was 52.3·10^3^ ± 6.0·10^3^ pps during normal walking, thus statistically different (*t*_(7)_ = 3.30, P = 0.01) from the ensemble estimated afferent activity of 32.9·10^3^ ± 18.7·10^3^ pps estimated during hybrid walking (Fig 3C).

### Relation between crossed responses and contralateral muscle estimated afferent activity during normal walking

**Fig 6:**
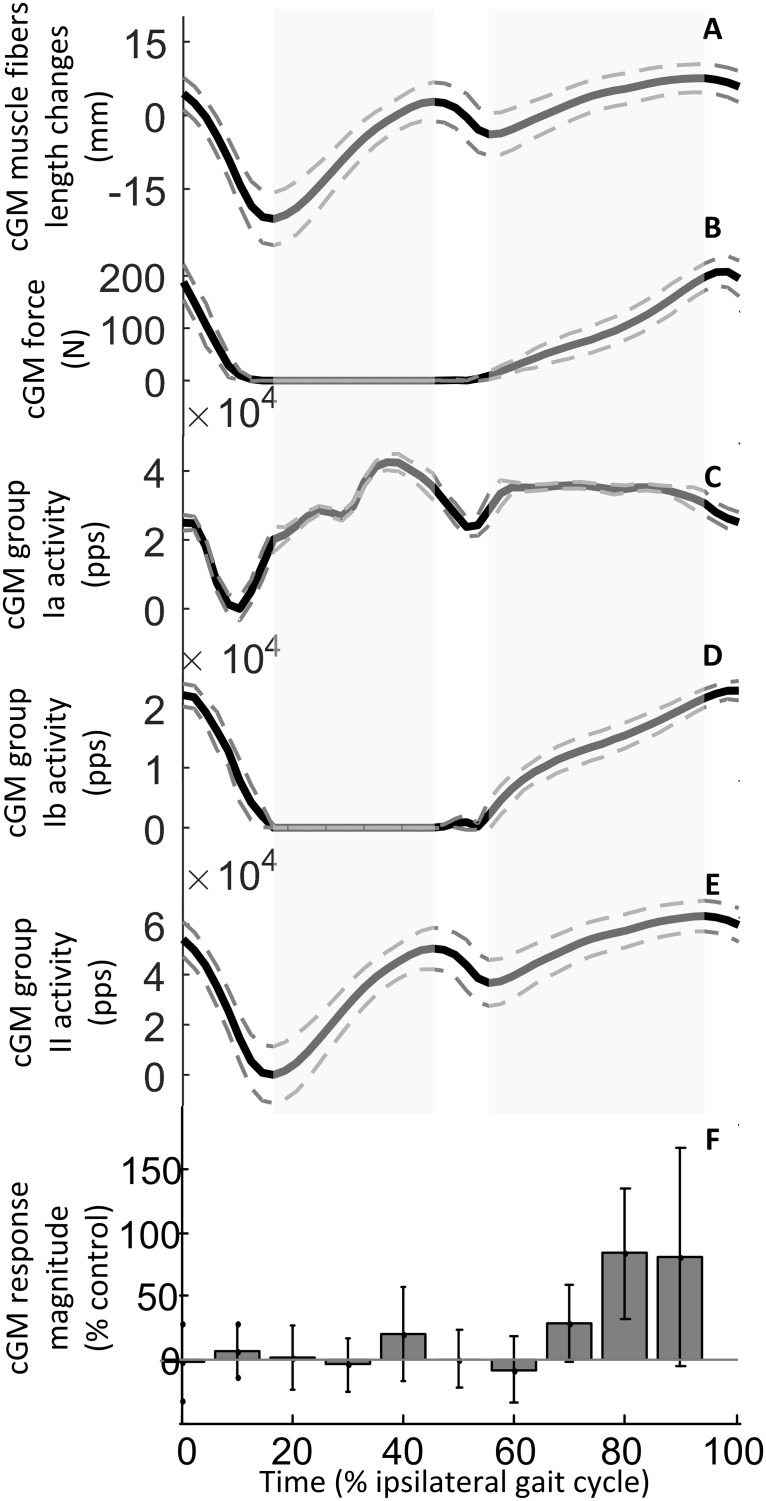
Modulation of estimated afferent feedback activity and SLCR in cGM during walking. Estimated muscle fascicles length changes (A) muscle force (B), and ensemble group Ia (C), Ib (D) and II (E) afferent activity estimated for cGM (data form 8 subjects) are shown to allow a comparison with the modulation of the short-latency crossed responses in cGM (F, data from 12 subjects) during a normal ipsilateral walking gait cycle. The full gray line and the dashed lines in A represent respectively the average and the standard deviation of the estimated values across all subjects. The state of stretch of the muscle are indicated by the gray areas. The thick black line and gray dashed lines in B, C, D and E represent respectively the mean estimated value of cGM muscle force (B) and ensemble afferent activity across all subjects (C, D, E). In F, the magnitude of the response elicited at different timings during the walking cycle is expressed (mean and SD) as a percentage of the control (no stimulation). Positive values indicate a facilitation and negative values indicate an inhibition of the muscle activation.

**Fig 7:**
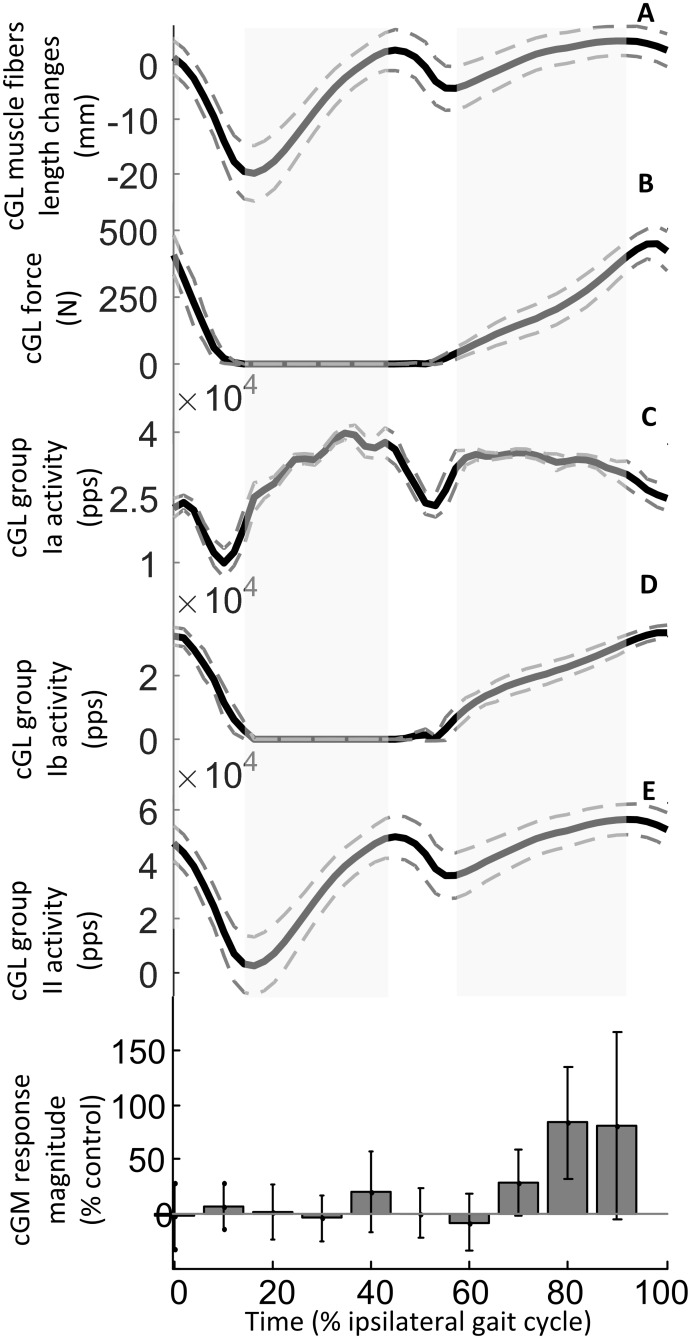
Modulation of cGL estimated afferent feedback activity and SLCR in cGM during walking. Estimated muscle fascicles length changes (A) muscle force (B), and ensemble group Ia (C), Ib (D) and II (E) afferent activity estimated for cGL (data form 8 subjects) are shown to allow a comparison with the modulation of the short-latency crossed responses in cGM (F, data from 12 subjects) during a normal ipsilateral walking gait cycle. The full gray line and the dashed lines in **A** represent respectively the average and the standard deviation of the estimated values across all subjects. The state of stretch of the muscle is indicated by the gray areas. The thick black line and gray dashed lines in **B**, **C**, **D** and **E** represent respectively the mean estimated value of cGM muscle force (**B**) and ensemble afferent activity across all subjects (**C**, **D**, **E**). In **F**, the magnitude of the response elicited at different timings during the walking cycle is expressed (mean and SD) as a percentage of the control (no stimulation). Positive values indicate a facilitation and negative values indicate an inhibition of the muscle activation.

In the current paper the modulation of the magnitude of short-latency responses in cGM was quantified (Figs 6F and 7F). While the electrical stimulation did not produce significant responses during the ipsilateral stance phase (P > 0.05), significant SLCRs were elicited in the cGM at 70% (t_(11)_ = 3.25, P = 0.008), 80% (t_(11)_ = 5.61, P < 0.001) and 90% (t_(11)_ = 3.28, P = 0.007) of the ipsilateral gait cycle. As reported previously for the cGL [15], the strongest facilitation occurred at 80% of the gait cycle with a mean amplitude of 183.63 ± 51.61% of the control signal. At this time during the gait cycle, the responses in the cGM had a latency of 58.3 ± 15.9 ms and duration of 36.6 ± 20.3 ms. The peak of the response occurred at 75.5 ± 11.1 ms after the stimulation, confirming that the time window for the quantification of the response was correctly selected. The presence of a modulation of the magnitude of the response during the gait cycle was also confirmed by the one-way Anova (*F*_(2.76)_ = 1.22, P < 0.01). Post-hoc comparisons revealed that, although the magnitude of the SLCRs elicited at 80 and 90% of the gait cycle did not significantly differ (P = 0.92), these were different from the responses elicited during the rest of the gait cycle (P < 0.05). In addition, the SLCR elicited at 70% of the gait cycle differed from that elicited at 60% (P = 0.005) but not from the others (P > 0.05), apart those at 80 and 90% of the gait cycle.

Fig 6 shows the muscle fascicles length changes (A), muscle force (B), group Ia (C), group Ib (D) and group II (E) estimated afferent outputs for the cGM (S4 file) and the modulation for the response in the same muscle (F) during a normal ipsilateral walking gait cycle. Fig 7 displays the muscle fascicles length changes (A), muscle force (B), group Ia (C), group Ib (D) and group II (E) estimated afferent outputs for the cGL (S4 file) in comparison with the modulation of the response in cGM (F) during a normal ipsilateral walking gait cycle. These results show that the values obtained with the described model, such as estimated muscle fascicle length changes and gastrocnemius lateralis muscle force amplitude are consistent with those reported by recent studies for human locomotion [28,39,40].

**Fig 8:**
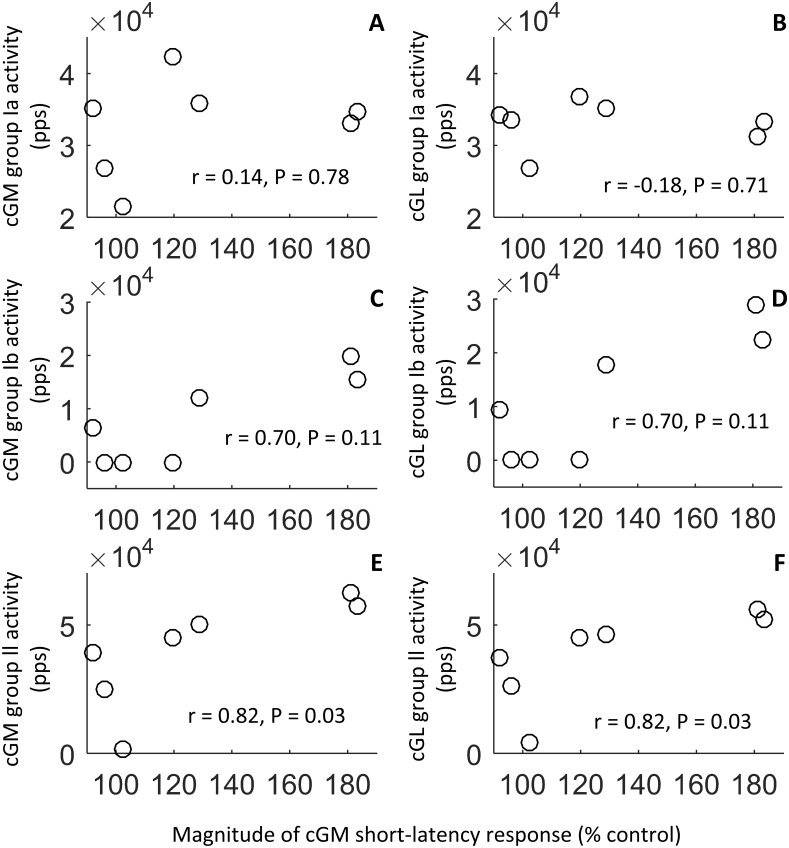
Correlations between the estimated ensemble afferent activity of cGM and cGL and the magnitude of the crossed responses elicited in cGM during the state of stretch of the muscle. No significant correlation was found between the magnitude of the cGM responses and group Ia afferent output of the same muscle (A) (P = 0.78, r = 0.14) and of cGL (B) (P = 0.71, r = -0.18). Similarly, no significant correlation (in both cases P = 0.11, r = 0.70) was observed between the magnitude of the responses and the ensemble group Ib activity of cGM (C) or of cGL (D). A significant correlation was instead observed between the magnitude of the cGM responses and ensemble group II afferent activity of cGM (E) and of cGL (F) (in both cases P = 0.03, r = 0.82).

Since animal studies suggest that crossed flexion is only evoked when the flexor muscles are stretched, otherwise a crossed extension would be evoked, only afferent activity associated with a state of stretch were compared to the magnitude of the response elicited in cGM; the state of stretch of the muscle, indicated by the gray zones (Figs 6 and 7), was observed at 20, 30, 40, 60, 70, 80, 90% of the gait cycle. A significant correlation was observed between the magnitude of the cGM responses and the group II afferent estimated activity of cGM and cGL (in both cases P = 0.03, r = 0.82). No significant correlation was instead found between the magnitude of the cGM responses and group Ia ensemble estimated afferent activity of the same muscle (P = 0.78, r = 0.14) nor between the cGM responses and group Ia ensemble estimated afferent activity of cGL (P = 0.71, r = -0.18). Similarly, there was no significant correlation between the magnitude of the cGM responses and group Ib ensemble estimated afferent activity for cGM and cGL (in both cases P = 0.11, r = 0.70) (Fig 8).

## Discussion

The current study investigated how sensory feedback is integrated in the control of interlimb coordination focusing on the contribution of contralateral muscle afferents to the SLCR and crossed reflex reversal in the human gastrocnemius muscles. A mathematical model based on inverse dynamics was adopted to estimate muscle spindles and GTO ensemble afferent activity. The estimated afferent activity during the gait cycle was then compared with the modulation of the SLCR in cGM. The results of the current study show that the modulation of the SLCR was correlated with the estimated ensemble group II afferent activity of both cGM and cGL. Thus, contralateral afferent feedback provided by muscle spindles is likely involved in the neural mechanism generating the SLCR. Moreover, in order to investigate whether a different afferent activity is the reason behind the reversal in the response observed between normal walking at 80% of the gait cycle and hybrid walking at 50% of the gait cycle [15], the estimated contralateral afferent activity in these two conditions was compared. Group Ib and II estimated activity differed when opposite responses have been previously observed in cGL [15]. Thus, the activity of group II fibers, likely contributes to the modulation of SLCR during normal walking, and is likely responsible for the observed reversal in response between normal and hybrid walking.

### Possible pathways and neural mechanism of the crossed response

Ipsilateral muscle afferents are the likeliest source for the short-latency responses in cGL during normal walking [15]. The results of the current study suggest however that contralateral muscle afferents also play a significant role in the SLCR (Fig 1).

In animal research, the main factor determining whether the crossed response produced by an ipsilateral non-specific perturbation results in extension or flexion was the position of the contralateral limb [4–7], with an excitatory response elicited in the muscle that is more stretched [4,5]. The results of the current study suggest that in humans, a similar mechanism might exist as ipsilateral stimuli produce a crossed facilitation in the cGL when the contralateral knee is extended and muscle fascicles are stretched; however, an inhibition is elicited when fascicles are shortened. Animal studies suggest that the response pattern is due to an ensemble action from the muscles of the leg [6,7]. In the current study, the responses in cGM were correlated with estimated secondary spindle activity of the same muscle and of cGL.

The SLCR might therefore depend on autogenic feedback from the same muscle, but also on heterogenic feedback from other muscles of the same limb. The existence of these ipsilateral feedback pathways has been reported to exist in humans and cats [41–43]. Evidence indicates the existence of two classes of heterogenic short-latency feedback mechanisms distributed according to architectural features of the musculoskeletal system [42,44,45]. Heterogenic length feedback leads to excitation of synergistic muscles crossing the same joint [44] and to inhibition of antagonistic muscles [45,46]. This is likely mediated by group Ia afferents, although a role for group II afferents is not excluded [45]. Heterogenic force related feedback is inhibitory and, although inhibition during locomotion is often replaced by excitation [47,48], it is unlikely that this feedback contributes to the SLCR as this feedback has been shown to extend from gastrocnemius muscles to SOL but not between GL and GM [45,49]. This force related feedback seems to be mediated by group Ib fibers and no correlation was observed between the crossed responses and group Ib activity in the current study.

The results thus suggest that afferent activity from group II fibers contributes to the modulation of the response. Although not directly supported by the results of the current study, the hypothesis that group II afferent activity plays a major role in the modulation of the SLCR is sustained by the observation that group I afferents appear to be suppressed [50] during gait probably through presynaptic inhibition of signal transmission from primary afferents [51,52]. Monosynaptic stretch reflexes can functionally destabilize posture [53], and since complex reactions are needed to compensate for instability during gait [50,54], an adequate polysynaptic response with a slightly longer delay might be more adequate [55]. Nevertheless, group Ia afferents contribute to reflex responses when an unexpected perturbation occurs [56].

For the reasons above, we suggest that length related feedback has a main contribution to the modulation of SLCR. This kind of feedback has been suggested to regulate joint stiffness, enhance force output during stance [57–59], strengthen the coupling between the ankle and knee joints [44], and contribute to the coordination of muscles during responses to postural disturbances [45,49]. Evidence in man [54] and cats [60] indicates that functionally appropriated EMG responses in the leg may be mediated predominantly by input from group II afferents [61]. The biomechanical information provided by muscle spindles, together with the high conduction velocity of these afferents, indicate that these receptors are suitable to provide the fast, relevant information needed for corrective responses during postural disturbances [62]. The larger response in the cGM shown in the current study compared to the response elicited in cGL [15], 183% and 138% of the control respectively, may be due to the larger numbers of spindle receptors in the former [63], which would produce a bigger autogenic feedback in cGM.

The current study did not investigate the contribution to crossed responses of contralateral cutaneous feedback which regulate the ongoing locomotor output during walking [64,65]. Thus, the contribution of these afferents cannot be excluded.

### Functional implications

Sensory information signaling whether the contralateral leg is prepared to sustain the body weight is essential in order to generate an appropriate crossed response. Indeed, the SLCR in different muscles is modulated during locomotion (current study, [13,15]) and a reversal is observed when an opposite reaction is required [15]. The most frequently observed crossed response in animals is an extension as a result of an ipsilateral stimulus unless such a response would be inappropriate, as when in the walking cat the contralateral leg is not in a position to sustain weight [10]. Such a condition, which occurs at the end of the ipsilateral swing phase when the contralateral limb is fully extended, would be signaled by the stretch of the flexor muscles [7]. In humans, at the end of swing phase, the contralateral leg is pushing off and continues to bear all body weight while the ipsilateral leg might not be ready for the phase transition. When a stimulation is delivered at this time of the gait cycle, afferent feedback from the contralateral leg, i.e. muscle spindles activity indicating the stretch of the gastrocnemii, signals the status (extension) of the contralateral knee, and contributes to generate a facilitation in this muscle group, enhancing the muscle stiffness and strengthening the coupling between ankle and knee [44].

### Methodological considerations

A direct measure of the overall afferent activity from muscles is not currently possible in the intact human; however, we propose that the estimate obtained with the method described in the current study i.e. taking the tendon compliance into consideration, is a better indicator of this parameter than other mathematical methods adopted in the past, based on estimation of changes in muscle tendon unit length instead of fascicle length changes [18,66]. The accuracy of the fascicle length changes estimation depends on the accuracy of the parameters selected for the model, such as the Young's modulus and tendon cross sectional area. We have made an effort to improve these model parameters with respect to age and gender of the population investigated. Since contradictory results can be found in the literature about a possible uniform fascicle behavior within a muscle [28,67,68] pennation angle values were selected from data reported in the literature for the central region of the muscles, as the EMG responses were recorded from this area. Moreover, although the muscle spindle model is derived from the cat triceps surae, the stretch sensitivity of human spindles at low velocities, as during walking, is similar to those of cats [69]. The results obtained with the described model are however consistent with those reported by recent studies for human locomotion: the modulation of the estimated muscle fascicle length changes (Figs 6A, 6B and 7A and 7B) is in accord with those displayed by [28,39] while muscle force amplitude is consistent with that reported by [40], with a maximum force exerted during walking by the gastrocnemius lateralis being just above 200 N.

An alternative to the estimation of muscle fascicle length through the inverse dynamics model here adopted would have been the use of ultrasound scanning. This method, however, do not provide a golden standard, due to the variety of approaches adopted by different research groups that result in large variability of the values reported [20]. Therefore, we assume that using the tendon model [17] to obtain muscle fascicle length changes gives an equally good and qualified estimate. Although advancement in the technique allow the observation of lengthening and shortening of fascicles in vivo during walking [28,70,71], such a technique raises some significant issues. First of all, the measurements are two dimensional, and are therefore unable to account for movement in additional plans [72] that could affect measurement of fascicle length and pennation angle. The introduction of 3D ultrasound may be able to offer a theoretical solution to this problem; however this technique cannot be applied yet to the study of movement. The use of the ultrasound probe constitutes another important issue for dynamic movements, since a cast and additional strapping are necessary to attach the probe to the skin. Although the structure is not usually of significant weight (~130g), the cable to which the probe is attached can be burdensome during locomotion [70]. For these reasons, a mathematical model has been preferred over the use of ultrasound scanning techniques.

## Conclusion

SLCR responses seem to contribute to dynamic stability. The current study provides evidence that during human walking, when the natural progression of one limb is threatened, sensory feedback from the contralateral limb, and not only from the one that has been perturbed, is essential to generate a proper reaction. These results underlines that therapeutics approaches aimed at increasing length related afferent feedback could be beneficial for improving dynamic balance of patients with locomotor disability. The current study provides therefore new insight into the neural mechanism behind interlimb coordination during human locomotion, with important implications for our understanding of which aspects (such as on methods to increase length related afferent feedback), the rehabilitation programs and the future research should be focusing on, in order to optimize locomotor recovery in patient populations.

## Supporting Information

S1 file Kinetic and kinematic data used to calculate the net ankle moment during normal walking. The file contains the data shown in Fig 4. All data (ankle and knee joints angles, vertical component of the GRF and estimated net ankle moment for the contralateral leg) represent an average gait cycle for one subject.
(XLSX)

S2 file Ratio between the pennation angle of gastrocnemius medialis and lateralis. The file contains data derived from [29], which was used in the current study to evaluate differences in cGM/cGL pennation ratio at different knee (0° and 45°) and ankle positions (-15°, 0°, 15°, 30°), and between active and passive conditions.
(XLSX)

S3 file Differences in estimated afferent activity between normal and hybrid walking. The file contains the values of the estimated ensemble activity for group Ia, Ib and II cGL’s afferent fibers at 80% of the normal walking ipsilateral gait cycle and at 50% of the hybrid walking ipsilateral gait cycle for 8 subjects. This data is shown in Fig 5.
(XLSX)

S4 file Modulation of cGM and cGL estimated afferent activity during walking. The file contains data for the estimated muscle fascicles length changes, muscle force, and ensemble group Ia, Ib and II afferent activity of cGM and cGL for 8 subjects during a normal ipsilateral walking gait cycle. This data is shown in Figs 6 and 7.
(XLSX)

S5 file Average estimated afferents activity during lengthening. The file contains the values of the estimated ensemble activity for group Ia, Ib and II afferent fibers of cGL and cGM during the state of stretch of the muscles (at 20, 30, 40, 60, 70, 80 and 90% of the ipsilateral gait cycle). This data was used in Fig 8.
(XLSX)
